# Self-assembly of Carbon Vacancies in Sub-stoichiometric ZrC_1−*x*_

**DOI:** 10.1038/srep18098

**Published:** 2015-12-15

**Authors:** Yanhui Zhang, Bin Liu, Jingyang Wang

**Affiliations:** 1 High-performance Ceramics Division, Shenyang National Laboratory for Materials Science, Institute of Metal Research, Chinese Academy of Sciences, Shenyang 110016, China; 2 School of Materials Science and Engineering, Shanghai University, Shanghai 200444, China

## Abstract

Sub-stoichiometric interstitial compounds, including binary transition metal carbides (MC_1−x_), maintain structural stability even if they accommodate abundant anion vacancies. This unique character endows them with variable-composition, diverse-configuration and controllable-performance through composition and structure design. Herein, the evolution of carbon vacancy (V_C_) configuration in sub-stoichiometric ZrC_1−*x*_ is investigated by combining the cluster expansion method and first-principles calculations. We report the interesting self-assembly of V_C_s and the fingerprint V_C_ configuration (V_C_ triplet constructed by 3^rd^ nearest neighboring vacancies) in all the low energy structures of ZrC_1−*x*_. When V_C_ concentration is higher than the critical value of 0.5 (*x* > 0.5), the 2^nd^ nearest neighboring V_C_ configurations with strongly repulsive interaction inevitably appear, and meanwhile, the system energy (or formation enthalpy) of ZrC_1−*x*_ increases sharply which suggests the material may lose phase stability. The present results clarify why ZrC_1−*x*_ bears a huge amount of V_C_s, tends towards V_C_ ordering, and retains stability up to a stoichiometry of *x* = 0.5.

Most covalent and ionic crystalline solids (daltonide) hold exact stoichiometry in order to keep translational symmetry and atomic coordination. In contrast, another group of compounds (berthollide, such as binary transition metal carbides and nitrides) maintain structural stability in a wide sub-stoichiometric range^1^,^2^,^3^. These materials have rock-salt crystal structure (B1) with C/N atoms locating at the octahedral interstitial sites of the *f.c.c.* sublattice constructed by transition metal atoms. The interstitial atomic sites are easy to form high concentration of C/N vacancies. For example, the concentration of carbon vacancies accommodated in TiC_1−x_ and ZrC_1−x_ is as high as 50%^4^,^5^. Both short-range ordering (SRO) and long-range ordering (LRO) of anion vacancy distribution are common in sub-stoichiometric materials^6^. Ordered phases can be fabricated by long-duration post annealing and rapid spark plasma (SPS) processing *etc.*^7^. These unique characters provide us the opportunity to succeed in defect engineering through modification of chemical composition and vacancy configuration. The related compounds were classified as ‘non-stoichiometric interstitial compound’^2^, and have attracted extensive interests since 1939^3^,^8^,^9^.

Zirconium carbide (ZrC) is a representative non-stoichiometric interstitial compound. It shows high hardness, high melting point, excellent high temperature thermal/mechanical properties, good wear and corrosion resistance, resistance to fission product attack and low neutron cross-section^10^,^11^,^12^. It is an important material as high-temperature component and hard coatings^5^,^13^, especially a promising candidate as nuclear fuel coating or cladding material^14^. Previous studies have found that carbon vacancies significantly affected its mechanical properties^15^,^16^, thermo-physical properties^12^ and microstructural stability under irradiation^17^. As a common phenomenon in MC_1−x_ carbides, the evolution of carbon vacancies dominates the longtime performance and is vital for understanding the high composition deviation.

During the last sixty years, a great number of theoretical and experimental progresses have been paced, in which the stable V_C_ configuration was the key concern. Reviewing the existing literature, we found that the conclusions were typically controversial. For instances, ordering phenomena in ZrC_0.51_^18^, ZrC_0.63_^19^, ZrC_0.67_^19^, ZrC_0.74_^19^ were claimed to be the same Zr_2_C superstructure at first. But later, Obata and Nakazawa proposed that the ordered phase in ZrC_0.70−0.75_ was actually Zr_4_C_3_, and there was no Zr_2_C ordered phase detected in ZrC_0.51_^20^. The knowledge was updated recently, *i.e.* the existence of Zr_2_C superstructure (*Fd-3m*)^7^ was firmly validated. To understand the mechanism of V_C_ ordering in transition metal carbides, Gusev proposed that the long-range interactions (probably the phonon subsystem) can account for LRO^21^. Novion held the opposite opinion that V_C_ ordering was dominated by short-range effects^3^. Up to now, the underlying mechanism pushing forward the evolution of V_C_ configurations is not fully understood.

In this paper, the energetics of ZrC_1−x_ at various V_C_ concentrations is studied using the state-of-the-art first principles and cluster expansion method. We report a fingerprint structural unit, namely the V_C_ triplet constructed by 3^rd^ nearest neighbor carbon vacancies in all the predicted low energy structures of ZrC_1−x_. The defective structure would lose stability when V_C_ concentration reaches over 0.5 and simultaneously, highly repulsive V_C_s with the 2^nd^ nearest neighbor coordination unavoidably appear. The results explain some longstanding puzzles in non-stoichiometric interstitial compounds, and this study may also shed lights on how to design or tailor the performances of promising transition carbides.

## Results

### Energetics of ZrC_1−x_

**Figure 1:**
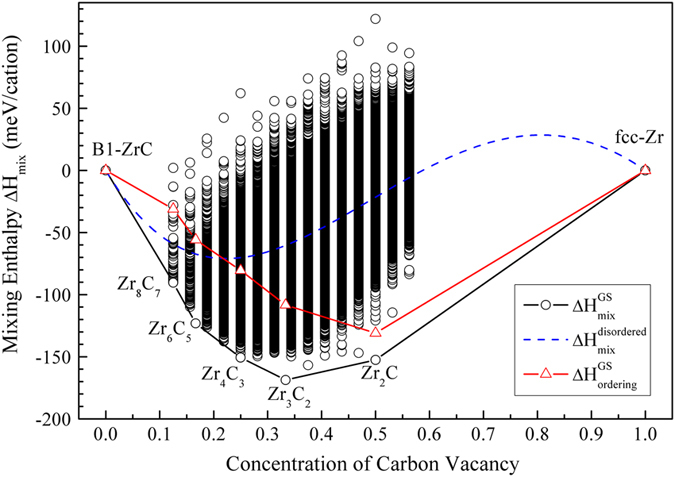
Mixing enthalpies (B1-ZrC and *f.c.c.* Zr as reference states) of ordered (the circles) and disordered (the dashed line, 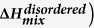 vacancy configurations with various compositions. The ground states are predicted as Zr_8_C_7_ (*P4*_*3*_*32*), Zr_6_C_5_ (*C2/m*), Zr_4_C_3_ (*C2/m*), Zr_3_C_2_ (*Fddd*) and Zr_2_C (*Fd-3m*) ordered phases.

The mixing enthalpies of diverse ZrC_1−x_ configurations (with reference to B1–ZrC and *f.c.c.* Zr, 0 ≤ x ≤ 0.5625), as illustrated by circles in Fig. 1, are predicted by cluster expansion method. There are five ground states (GSs), including ordered Zr_8_C_7_ (*P4*_*3*_*32*), Zr_6_C_5_ (*C2/m*), Zr_4_C_3_ (*C2/m*), Zr_3_C_2_ (*Fddd*) and Zr_2_C (*Fd-3m*) phases, that restrict the lower bound of mixing enthalpies (see the GS envelope line in Fig. 1, *i.e.* the black curve with circles). These GSs are the configurations with the lowest energy at each composition and will not undergo phase separation into disproportionation products. It would be stated that the ground states of Zr_8_C_7_ (*P4*_*3*_*32*), Zr_4_C_3_ (*C2/m*) and Zr_2_C (*Fd-3m*) are found by exhaustive search in simulation box of 2×2×2 supercell (32 Zr sites); and Zr_6_C_5_ (*C2/m*) and Zr_3_C_2_ (*Fddd*) are disclosed by simulated annealing method in large configuration space with up to 1726 Zr sites (12×12×12 supercell). By this way, the predicted GS structure at Zr_6_C_5_ (*C2/m*) is isotypic with that of Ti_6_C_5_ (*C2/m*)^22^, and the one at Zr_3_C_2_ (*Fddd*) is isotypic with that of Sc_2_S_3_ (*Fddd*)^23^ (its energy is 4 meV/cation lower than that isotypic with Ti_3_C_2_ (*C2/m*) predicted in ref. 22). In experiments, only the ordered Zr_2_C (*Fd-3m*) phase was characterized by selected area electron diffraction^7^ and neutron diffraction^18^ methods. Besides, Obata and Nakazawa observed superlattice lines in annealed ZrC_0.7_ by X-ray diffraction^20^. They proposed the existence of ordered Zr_4_C_3_ phase, but did not present the crystal structure. Although predicted Zr_8_C_7_ (*P4*_*3*_*32*), Zr_6_C_5_ (*C2/m*) and Zr_3_C_2_ (*Fddd*) phases were not found before, their isotypes, V_8_C_7_ (*P4*_*3*_*32*)^9^, Ti_6_C_5_ (*C2/m*)^22^ and Sc_2_S_3_ (*Fddd*)^23^, have been reported.

The energetics of sub-stoichiometric ZrC_1−x_ presents more information for vacancy tolerance and ordering capability. Firstly, ZrC_1−x_ displays significant tolerance to high concentration of V_C_s. The mixing enthalpy of ZrC_1−x_ with random V_C_ distribution is shown by the dashed curve in Fig. 1. The predicted mixing enthalpies retain negative in the composition range of 0 < *x* < 0.59. This result suggests that sub-stoichiometric ZrC_1−x_ phases with a huge amount of vacancies are energetically favorable. Otherwise, sub-stoichiometric ZrC_1−x_ with positive mixing enthalpy would spontaneously decompose into *f.c.c.* Zr and B1-ZrC competition phases. Furthermore, sub-stoichiometric ZrC_1−x_ demonstrates obvious tendency of V_C_ ordering because numerous V_C_ configurations have lower mixing enthalpies than the disordered V_C_ distribution in Fig. 1. The ordering enthalpies of GSs 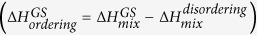, as represented by the triangles in Fig. 1, are always negative. Meanwhile, with increase of V_C_ concentration, the ordering enthalpy continuously decreases and reaches the minimum value at around *x* ~ 0.5. This suggests the fact that the higher the V_C_ concentration, the stronger the ordering tendency of V_C_s, and in addition, Zr_2_C has the most obviously ordering tendency in all studied sub-stoichiometric ZrC_1−x_ structures. We expect that ordered Zr_2_C should be the most possible ordered phase synthesized in experiments. This result is consistent with the discovery of ordered Zr_2_C in experiments^7^,^18^. Besides, the calculated ordering enthalpies of Zr_8_C_7_ and Zr_6_C_5_ are –30 meV/cation and –55 meV/cation, respectively, which are comparable to that of V_8_C_7_ and V_6_C_5_ ordered phases (around –20 meV/cation)^3^. Because all the GS structures have negative ordering enthalpies, other predicted GS phases would be fabricated by careful controlling of experimental conditions.

### Vacancy configurations

**Figure 2:**
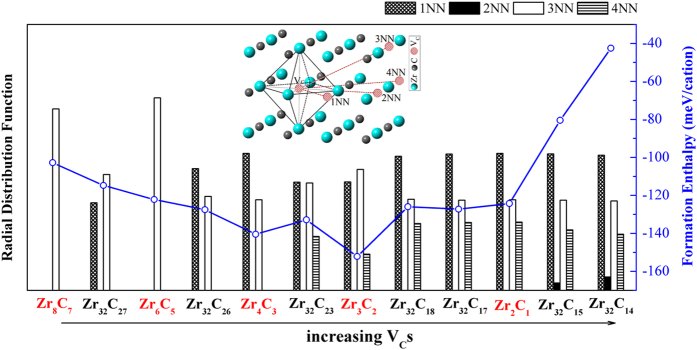
Radial distribution function of nearest neighboring (NN) V_C_ pairs in low energy structures (red labels for GSs, black labels for near GSs), as well as the variety of their formation enthalpies. The inset shows the V_C_ sites in 1NN, 2NN, 3NN and 4NN V_C_ pairs along {110}, {200}, {211} and {220} crystal directions, respectively.

Besides the predicted ground states, the structural characteristics of low energy configurations between neighboring GSs are also important to depict the evolution features of V_C_s in sub-stoichiomitric ZrC_1−x_. Figure 2 illustrates the radius distribution function of V_C_ pairs in selected low energy structures with various compositions. V_C_ configurations are displayed by the presence or absence of certain neighboring V_C_ pairs, including 1NN (1^st^ nearest neighboring), 2NN (2^nd^ nearest neighboring), 3NN (3^rd^ nearest neighboring), and 4NN (4^th^ nearest neighboring) V_C_ pairs. It is striking to notice that 3NN V_C_ pair is found in all structures, especially only 3NN V_C_ pair appears in the GS structures of Zr_8_C_7_ and Zr_6_C_5_ which have low V_C_ concentrations. When V_C_ concentration increases, 1NN V_C_ pair subsequently presents in the GS of Zr_4_C_3_, then 4NN V_C_ pair emerges in the GSs of Zr_3_C_2_ and Zr_2_C. Besides, 1NN V_C_ pair is also found in the low energy structures near the GSs, such as Zr_32_C_27_ and Zr_32_C_26_. At higher V_C_ concentration, 4NN V_C_ pair is identified in low energy structures near the GSs, such as Zr_32_C_23_, Zr_32_C_18_, and Zr_32_C_17_. When V_C_ concentration is higher than 50%, 2NN V_C_ pair inevitably arises, such as Zr_32_C_15_ and Zr_32_C_14_. Accordingly, their formation energies increase sharply with the presence of 2NN V_C_ coordination.

Figure 2 clearly displays the interesting characteristics of V_C_ configurations: most frequently observed 3NN configuration, available 1NN and 4NN configurations, as well as the unfavorable or forbidden 2NN configuration. It is important to disclose the global evolution characteristics of V_C_s in binary transition metal carbides. Indeed, it was both experimentally and theoretically found that the vacancies in nonstoichiometric interstitial carbides preferred the 3NN shell and excluded the 2NN shells^3^,^22^,^24^, which was consistent with our calculation results. Hart *et al.* investigated the vacancy distribution in low energy structures of TiC_1−x_ using cluster expansion method. Although they did not mention the evolution of V_C_ configurations at various concentrations, carbon vacancies were found arranging themselves in [112] rows^25^, which was along the crystal direction of 3NN V_C_ pairs, and 2NN configurations gradually disappeared during annealing or ordering.

**Figure 3:**
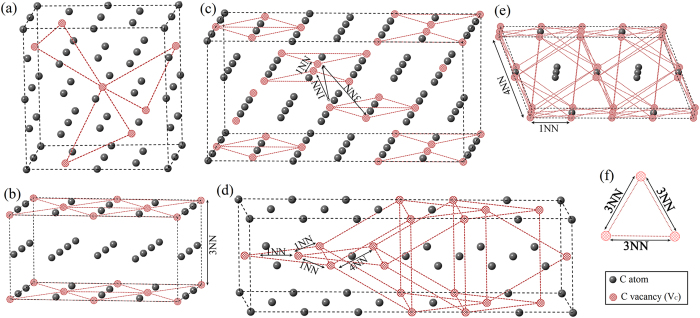
Vacancy configurations in C-sublattice of (**a**) Zr_8_C_7_ (*P4*_*3*_*32*), (**b**) Zr_6_C_5_ (*C2/m*), (**c**) Zr_4_C_3_ (*C2/m*), (**d**) Zr_3_C_2_ (*Fddd*) and (**e**) Zr_2_C (*Fd-3m*), with significant character of (**f**) 3NN V_C_ triplet.

We expect that there would be inherent local V_C_ configurations commonly appearing in the low energy structures because their mixing enthalpies are so close to the GS envelope line. In ZrC_1−x_ with stoichiometry near ordered GS phases (around compositions of Zr_2_C, Zr_4_C_3_, Zr_3_C_2_ *etc.*), it would be unusual for any abrupt change of V_C_ patterns. After careful analysis of V_C_ configurations, the common configuration unit, *i.e.* the 3NN V_C_ triplet, is identified. Figure 3 illustrates the overall features of V_C_ distributions in C-sublattice. In Zr_8_C_7_ (*P4*_*3*_*32*) and Zr_6_C_5_ (*C2/m*) as shown in Fig. 3(a,b), respectively, neighboring V_C_s only occupy 3NN sites and form the 3NN V_C_ triplets. The schematic of 3NN V_C_ triplet is displayed in Fig. 3(f), which is an equilateral triangle with its side length restricted by 3NN coordination. Although it was known that the preference of occupying 3NN V_C_ shell was typical in ordered structures of sub-stoichiometric transition metal carbides and nitrides, the significance of V_C_ triplet configuration was not reported before. We find that the 3NN V_C_ triplet prevails in all the low energy structures like a fingerprint V_C_ configuration.

The 3NN V_C_ triplets are corner-shared in Zr_8_C_7_ (*P4*_*3*_*32*) (as shown in Fig. 3(a)), and edge-shared in Zr_6_C_5_ (*C2/m*) (as shown in Fig. 3(b)). In Zr_6_C_5_ (*C2/m*), one defective carbon layer (filled with 2/3 carbon atoms) and its neighboring perfect carbon layer stack alternatively along < 211 > _B1_ crystal direction. When V_C_ concentration is higher than 1/6, V_C_s could not be accommodated by occupying the sites restricted by pure 3NN coordination. Neighboring 3NN V_C_ triplets adjust their relative positions and bring out the 1NN V_C_ configuration to host more V_C_s, as shown for Zr_4_C_3_ (*C2/m*) in Fig. 3(c). The 3NN V_C_ triplets are predominant in the {111}_B1_ carbon layers and stack along < 211 > _B1_ crystal direction. With more V_C_s incorporated in Zr_3_C_2_ (*Fddd*), 3NN V_C_ triplets link adjacent defective carbon layers, as shown in Fig. 3(d). Additionally, self-assembling of 3NN V_C_ triplets generates the 1NN and 4NN V_C_ configurations. For Zr_2_C (*Fd-3m*) shown in Fig. 3(e), neighboring carbon layers have 1/3 and 2/3 C-sublattice sites occupied by V_C_s, respectively, in which crowded 3NN V_C_ triplets are orthocenter overlapped. It is noted that every carbon atom in Zr_2_C (*Fd-3m*) is surrounded by overlapped 3NN V_C_ triplets. These 3NN V_C_ triplets are completely coordinated by 1NN and 4NN V_C_ configurations. Changing any V_C_ site or forming one more V_C_ will introduce the 2NN coordination. Besides these GS structures, remarkable 3NN V_C_ triplets are also found as the common configuration unit in near GS configurations, such as Zr_32_C_25_ (*C2*) and Zr_32_C_23_ (*R-3m*).

### Short-range interactions

The above results illustrate the self-assembling of V_C_ configuration and feature the fingerprint configuration of 3NN V_C_ triplet in defective ZrC_1−x_. It is speculated that the redistribution of electrons around vacancies^22^,^26^,^27^ would affect the short-range interactions among V_C_s by altering the M-C bonds, which would be the important driven force pushing forward the local ordering pattern of V_C_s. Therefore, the interaction (or binding) energies of various V_C_ clusters, and their correlations with the evolution of V_C_ configurations, are studied here. Using the 3×3×3 supercell with 108 Zr sites, the interaction energies of V_C_ pair and triplet are calculated by the following equations^28^:


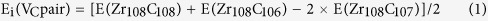



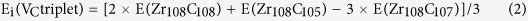


where the first and last terms on the right side are the total energies of perfect ZrC and the supercell containing an isolated V_C_, respectively; and the second items in equations (1) and (2) stand for the total energies of the supercells containing various V_C_ pairs and the 3NN V_C_ triplet, respectively. The derived interaction energy indicates the thermal stability of various vacancy clusters relative to isolated V_C_s with infinite distance. and determines whether isolated vacancies would aggregate together to form certain vacancy configuration.

**Figure 4:**
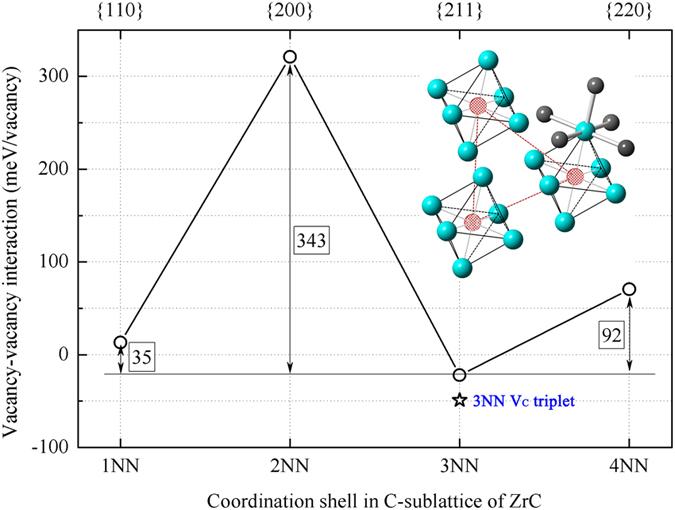


Figure 4 plots the interaction energies of various V_C_ pairs and the 3NN V_C_ triplet. For V_C_ pairs, interaction energies increase in the order of *E*_*i*_(3NN) < 0 < *E*_*i*_(1NN) < *E*_*i*_(4NN)<<*E*_*i*_(2NN), which agrees with that reported by Razumovskiy *et al.*^29^,^30^. The interaction energy of 3NN V_C_ triplet yields –49 meV/vacancy or −147 meV/triplet, which is lower than that of 3NN V_C_ pair (–22 meV/vacancy or −44 meV/pair). Therefore, the formation of 3NN V_C_ triplet benefits to lower the energy of defective structure. The inset in Fig. 4 sketches neighboring Zr_6_C octahedra with a 3NN V_C_ triplet. The C atoms in octahedra are removed to produce V_C_s, and the 3NN V_C_ triplet configuration ensures each Zr atom coordinated with only one V_C_. Therefore, the carbon coordination of Zr atom is minimally disturbed, and thereby the bonding feature is least affected. Meanwhile, the self-assembling of V_C_s allows short-range ordering by coordinating the most preferred V_C_ configuration, *i.e.* 3NN V_C_ triplet. Local ordering in the configuration of 3NN V_C_ triplets would be universal in ZrC_1−x_ at any composition. In elastic diffuse neutron scattering experiments, ZrC_0.80_ and ZrC_0.64_ were identified with the same peak positions although they have different compositions^3^. This result suggests similar short-range ordering in the two compounds. If the short-range ordering covers the whole lattice and the defective structure satisfies a new symmetry at certain composition, then an ordered ZrC_1−x_ phase would be identified. All the results show that self-assembling of 3NN V_C_ triplets is the key factor to maintain phase stability of defective ZrC_1−x_ with high concentration of V_C_s, and to realize short-range and long-range ordering of V_C_s.

The interaction energy of 1NN V_C_ pair, 13 meV/vacancy, is slightly higher than zero but much lower than those of the 4NN and 2NN V_C_ pairs, which are 70 meV/vacancy and 321 meV/vacancy, respectively. The small value of 1NN V_C_ pair shows very weakly repulsive interaction. Therefore, 1NN V_C_ pair is the second preferred configuration in the low energy structures. With more V_C_s accommodated in GS structures of Zr_3_C_2_ and Zr_2_C, together with near GS structures Zr_32_C_23_, Zr_32_C_18_ and Zr_32_C_17_, both 1NN and 4NN V_C_ pairs present in order to efficiently coordinate neighboring 3NN V_C_ triplets. Therefore, the presences of 1NN and 4NN V_C_ configurations are helpful for balancing V_C_ concentration and thermal fluctuation.

The 2NN V_C_ pair has extremely high interaction energy (321 meV/vacancy), which means significantly repulsive interaction between V_C_s in 2NN configuration. Therefore, 2NN V_C_ pair is unfavorable or forbidden in defective ZrC_1−x_ and it does not show up in low energy structures when V_C_ concentration is lower than 50%. It’s noteworthy that the strongly repulsive interaction among 2NN configurations may prevent the formation of large-scale V_C_ clusters in sub-stoichiometric ZrC_1−x_. Unfortunately, 2NN coordination is inevitable when V_C_ concentration is higher than 50%. The appearance of 2NN configuration will abruptly increase the formation enthalpy (shown in Fig. 2) and therefore, the defective ZrC_1−x_ may undergo phase separation in such case. This might be the reason why the critical V_C_ concentration is limited around 50% in ZrC_1−x_.

The short-range interactions among vacancies could well explain the evolution feature of V_C_ configurations and the fingerprint configuration. The result indicates that these are the driven-force of V_C_ self-assembling, *i.e.* bringing down the system energy *via* the maximization of attractive 3NN configuration and the exclusion of strongly repulsive 2NN coordination, and simultaneously balancing 3NN V_C_ triplets through the moderately repulsive 1NN and 4NN interactions. Also, the short-range interactions among V_C_s would be more and more significant at high V_C_ concentration. It may provide hints on the origin of enhanced ordering tendency with increasing V_C_s.

### Electronic structures of ZrC_1−x_

**Figure 5:**
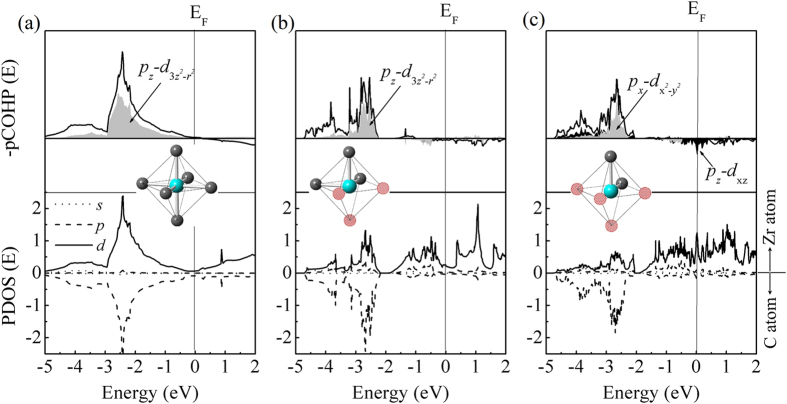
The projected density of states (PDOS, unit states/eV·atom) and the projected crystal orbital Hamilton population (pCOHP) of nearest-neighbor Zr-C interactions in (**a**) ZrC (*Fm-3m*), (**b**) Zr_2_C (*Fd-3m*) and (**c**) Zr_32_C_15_ (*R-3m*). The solid lines in pCOHP diagram stand for the sum of *pd* bands, while the shading zones denote the contributions from certain orbitals (No scale is given for the pCOHP analysis).

Projected density of states (PDOS) and projected crystal orbital Hamilton population (pCOHP)^31^ are illustrated in Fig. 5 to describe the Zr-C bonding characters in highly defective ZrC_1−x_. At high V_C_ concentration, the electronic structures of ZrC_1−x_ would be significantly affected by vacancy-vacancy interaction and/or vacancy ordering. This was verified by Pickett and Klein as they found complex differences between the calculated electronic structure of an isolated carbon vacancy in B1-NbC by the muffin-tin Green’s-function method and the experimental electronic structure of NbC_0.85_ from X-ray photoemission spectrum^32^. Therefore, we investigate the electronic structures of ordered Zr_2_C and Zr_16_C_15_ phases to understand the electronic structures of ZrC_1−x_ around the critical composition of *x* = 0.5. This would illustrate the mechanism of electronic redistribution at high V_C_ concentration.

Firstly, we study the bonding characters in perfect ZrC (B1) as shown in Fig. 5(a). In the perfect unit cell, Zr atom is octahedral coordinated by six C atoms with a high site symmetry (O_h_), which ensures good capability of band overlapping. The bonding region in PDOS shows a strong hybridization of C *p*- and Zr *d*-states, which promotes the formation of strong covalent Zr-C bond. Besides, the orbital-wise pCOHP analysis indicates that Zr-C bond is dominated by the *p-d* σ and π bonding with significant share of C(*p*_*z*_) and Zr 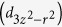 interactions. These bonding characters are consistent with other transition metal carbides like TiC^26^,^33^.

With removing of C atoms, *i.e.* the electron acceptors in ZrC_1−x_, the excess *d* electrons redistribute on electronic states in high energy level, as clearly shown in Fig. 5(b). For Zr_2_C at the critical concentration, the bonding states between –2.4 eV and –4.8 eV are mainly dominated by C(*p*_*z*_) and Zr 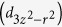, and the PDOSs locating from –1.9 eV to Fermi level (E_F_) correspond to the *d-d* bonding among Zr atoms. When the concentration of V_C_s is higher than 50%, for example Zr_32_C_15_ (*R-3m*) with inevitable 2NN V_C_ coordination, anti-bonding states originated from C(*p*_*z*_)–Zr(*d*_*xz*_) interactions obviously show up near E_F_ in Fig. 5(c). As a result, the formation enthalpy is quite high for ZrC_1−x_ with *x* > 0.5, which contains unfavorable 2NN V_C_ configuration.

## Discussion

We found neighboring V_C_s have totally different values of short-range interactions, which increase in the order of *E*_*i*_(3NN) < 0 < *E*_*i*_(1NN) < *E*_*i*_(4NN) << *E*_*i*_(2NN). It goes along with the evolution characteristics of V_C_ configurations. The moderately attractive and strongly repulsive interactions between V_C_s in 3NN and 2NN configurations, respectively, provide the driven-force of self-assembling of V_C_s. Meanwhile, 3NN V_C_ triplet is more stable than other V_C_ configurations, and it serves as the fingerprint block in low energy ZrC_1−x_ structures. At high V_C_ concentration, neighboring 3NN V_C_ triplets modify relative positions by coordinating 1NN and 4NN V_C_ configurations. The present results clearly demonstrate that ordering of V_C_s in ZrC_1−x_ is not an abrupt structural change. The local ordered configurations, *i.e.* 3NN V_C_ triplets, already present in low energy structures at any composition; and at special composition, long-range ordering spreads throughout the whole lattice and one could observe ordered phase with new space group. The underlying mechanism falls into the concept of self-assembling of V_C_s. Short-range interactions among V_C_s are the driven-force of local or long-range ordering; and the formation enthalpy is reduced *via* the generation of attractive 3NN interactions and the exclusion of strongly repulsive 2NN interactions.

It is crucially important to realize the local ordering of V_C_s and to maintain phase stability in highly sub-stoichiometric ZrC_1−x_. On one hand, local ordering of V_C_ configuration with 3NN triplets, instead of the disordered distribution, stabilizes the defective structures by minimally affecting bonding features. On the other hand, the strongly repulsive interaction between V_C_s in 2NN configuration weakens the formation of large scale vacancy clusters or voids as well. More encouragingly, the self-assembling of V_C_s provides us the opportunity to tailor overall properties through defect engineering. One exciting perspective is the improvement of radiation resistance of ZrC_1−x_ *via* the mechanism of vacancy mediated performance optimization. Local ordering of V_C_s benefits the accommodation, annihilation and recombination of radiation-induced C-related point defects. Therefore, ZrC_1−x_ with optimal composition may be promising for next generation nuclear fuel coating and cladding material. Another important prospect is to precisely tune mechanical and thermal properties through adjusting the chemical composition of ZrC_1−x_. In the near future, it demands extensive investigations on the effects of V_C_ concentrations and configurations on radiation resistance and thermo-physical properties.

We herein proposed the physical insight of the critical V_C_ concentration, *x* ~ 0.5, in ZrC_1−x_. At relatively low V_C_ concentration, excess *d* electrons redistribute on *d-d* bonding states after the removal of C atoms; whilst neighboring Zr-C bonding is not affected significantly. This mechanism is helpful to maintain the structural stability of defective ZrC_1−x_. When the V_C_ concentration is higher than the critical composition, 2NN V_C_ configuration inevitably appears which brings out anti-bonding states between Zr and C atomic interactions. The anti-bonding states lead to extremely high formation enthalpy of defective ZrC_1−x_ with 2NN V_C_ coordination and thereby, the ZrC_1−x_ compound loses stability and undergoes phase separation.

The current investigation suggests new clue for better understanding of the phase stability and defect engineering in non-stoichiometric interstitial compounds. Firstly, the phase stability and ordering mechanism in ZrC_1−x_, especially the discovery of self-assembling of 3NN triplets driven by short-range interactions among V_C_s, may be common in binary transition metal carbides. There are solid evidences on the similar V_C_ configurations in non-stoichiometric carbides, for instances, the isotopic structures of V_8_C_7_ (*P4*_*3*_*32*)^9^ and Ti_6_C_5_ (*C2/m*)^22^ with Zr_8_C_7_ (*P4*_*3*_*32*) and Zr_6_C_5_ (*C2/m*), respectively; the widespread occupation of 1NN and 3NN shells, but not 2NN shells in TiC_1−x_ and ZrC_1−x_^6^; as well as the same neutron diffuse scattering in TiC_0.5−0.6_, VC_0.75_ and NbC_1−x_^3^,^34^. This work presents hints to understand following puzzles, like why common characteristics of vacancy configurations exist and what are mechanisms of phase stability and V_C_ ordering in sub-stoichiometric binary transition metal carbides. Secondly, it needs thorough inquiry whether the present results could be extended to other sub-stoichiometric binary transition metal nitrides and oxides because short-range interactions among anion vacancies are possibly different. Various self-assembling mechanisms of anion vacancies may dominate the diversity of the ordering phenomena in sub-stiochiometric nitrides and oxides.

In summary, vacancy configurations and their evolution in sub-stoichiometric ZrC_1−x_ were investigated by combining first-principles calculations, cluster expansion and supercell methods. Firstly, the negative mixing enthalpy and the negative ordering enthalpy are direct energetic proofs of the tolerance and the ordering tendency of V_C_s in sub-stoichiometric ZrC_1−x_. Besides, the energetically preferred 3NN V_C_ triplet is the fingerprint structural unit in sub-stoichiometric ZrC_1−x_. To balance V_C_ concentration and thermal fluctuation, 1NN and 4NN V_C_ configurations with moderately repulsive interactions would show up. The tolerance of vacancies is limited at around 50% because the presence of unfavorable 2NN V_C_ coordination with strongly repulsive interaction energy would lead to structural instability or phase separation.

It’s noteworthy that the self-assembling of 3NN V_C_ triplets driven by short-range interactions is the fundamental essence of the phase stability, short-range and long-range ordering in sub-stoichiometric ZrC_1−x_. For the first time, we disclose the significant self-assembling mechanism of V_C_s in sub-stoichiometric ZrC_1−x_, which provides us the opportunity to tailor its properties, such as the radiation resistance, mechanical and thermal properties, through defect engineering.

## Methods

Sub-stoichiometric ZrC_1−x_ is taken as a pseudo “binary solid solution” consisting fully occupied Zr-sublattice and mixing of carbon atoms and vacancies at C-sublattice. This system can be handled by supercell^4^,^35^, order parameter functional method (OPF)^36^, coherent-potential approximation^37^, or cluster expansion (CE)^38^ methods. Three ordered phases, Zr_2_C, Zr_3_C_2_ and Zr_6_C_5_, have been predicted using OPF method^36^, but the results did not provide structural information. Theoretical calculations using supercell method^4^,^35^ has emphasized the importance of lattice relaxation effect and the vacancy distribution, however it may leave out important configurations because of limited trial structures. The CE method which is the choice here, considers the effects from short-range interactions and local relaxation, and therefore provides both the structural characteristics and corresponding structural energy with DFT (Density Functional Theory) accuracy^39^.

Within the CE approach, the configuration on C-sublattice is described by configurational pseudospin variable *σ*_i_ for lattice site *i*, which takes value +1 or −1 depending on the occupation of lattice site *i* by carbon atom or carbon vacancy. A particular arrangement of spins is called a configuration (or structure), which is represented by a vector {*σ*_i_} containing the value of configurational pseudospin variable for each site *i*. The energy of a given composition and configuration is expressed as^39^,^40^:


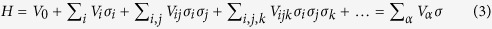


where *V*_*α*_ and *φ*_*α*_ are called the effective cluster interaction (ECIs) and the correlation function of cluster *α* (corresponding to a set of sites *i*: pairs, triplets, tetrahedral, *etc*.), respectively. For disordered phases, the energy is only composition (*x*) dependent^41^:


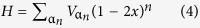


where *α*_*n*_ stands for one kind of n-body cluster.

A principal objective of the CE method is to evaluate unknown ECIs so that the configuration characters can be predicted with the accuracy of DFT calculations. Here, we determine the ECIs in the framework of the structure inversion method^42^,^43^,^44^. When energies of ordered structures are prepared from DFT calculations, the ECIs are determined by fitting a truncated form of equation (3) to the DFT energies using the least-squares technique. The accuracy of CE prediction is assessed by the leave-one-out and leave-many-out cross-validation (CV) score^42^,^43^,^44^. The set of clusters is optimized using genetic algorithm *via* minimizing the CV score. The DFT structures include initial selected random structures, low energy structures refined during construction of CE, and reported superstructures in non-stoichiometric interstitial compounds^9^,^45^. Finally, 25 clusters and 250 structures were selected to construct the CE formula. The average prediction error of the final CE is less than 4.3 meV/cation (per Zr/C-sublattice site). Thereafter, two types of calculations are used to search low energy structures (LESs) *via* the constructed CE. One is an exhaustive search by calculating the energies of all possible configurations in a finite-sized cell. The other is the simulation annealing (SA) method^46^ within a larger structural space. Here, exhaustive search was used for the supercell containing 32 or less Zr sites and the SA method was used for the supercell containing up to 1728 Zr sites. Detailed procedure of constructing CE can be found in ref. 47. In this work, CLUPAN code^42^,^48^ is used for the construction of CE and the searches of LESs.

First-principles calculations were done using VASP^49^ code, in which the projector augmented-wave (PAW) method^50^,^51^ within generalized gradient approximation^52^ was employed. The plane-wave basis cutoff and the *k*-mesh separation were set as 500 eV and 0.04 Å^−1^, respectively. Full structural relaxations (atomic positions and lattice constants) were performed until the energy difference converges to less than 10^−6^ eV.

## Additional Information

**How to cite this article**: Zhang, Y. *et al.* Self-assembly of Carbon Vacancies in Sub-stoichiometric ZrC_1-x_. *Sci. Rep.* **5**, 18098; doi: 10.1038/srep18098 (2015).
